# Sulfadiazine-induced crystalluria and non-oliguric renal failure in HIV-1 inaugural infection with presumed cerebral toxoplasmosis

**DOI:** 10.1590/2175-8239-JBN-2023-0151en

**Published:** 2024-05-10

**Authors:** Vasco Gaspar, Nuno Moreira Fonseca, Sara Lino

**Affiliations:** 1 Centro Hospital Universitário Lisboa Central, Departamento de Medicina Interna, Lisboa, Portugal.; 2 Universidade NOVA de Lisboa, Faculdade de Medicina NOVA, Lisboa, Portugal.; 3 Centro Hospitalar Universitário Lisboa Central, Hospital Curry Cabral, Departamento de Nefrologia, Lisboa, Portugal.; 4 Centro Hospitalar Universitário Lisboa Central, Hospital Curry Cabral, Departamento de Infecciologia, Lisboa, Portugal.

**Figure 1:**
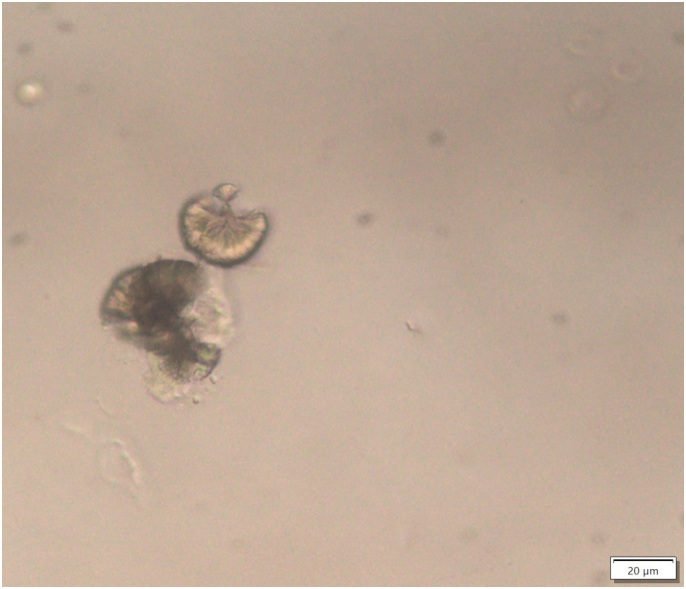
Sulfadiazine crystals have an amber color and radial striations (contrast phase microscopy, 400× magnification). Urinary analysis results – density: 1.008; pH: 5; proteins: 15 mg/dL; hemoglobin: 0.75 mg/dL; nitrites/glucose/ketones/bilirubin/urobilinogen: negative.

**Figure 2:**
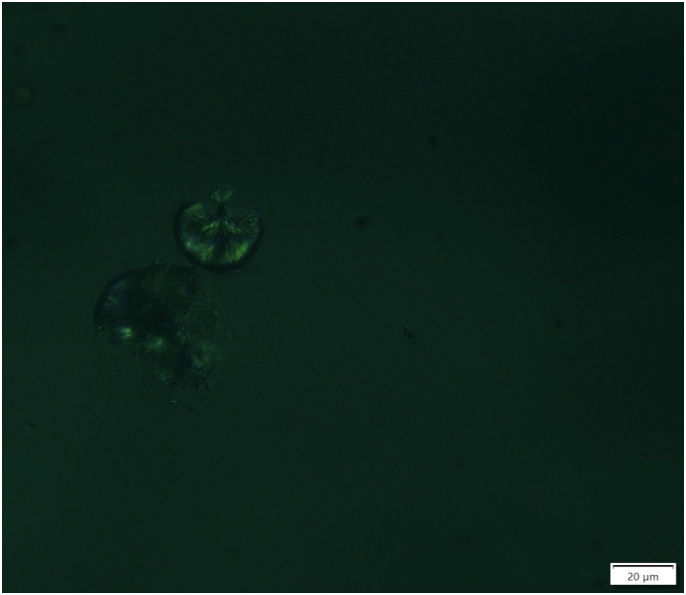
Sulfadiazine crystals are strongly birefringent under polarized light (polarized light, magnification 400×).

A 30-year-old woman with no previous medical history presented to the emergency department with a seizure and aggressive behavior. After extensive blood work and imaging studies, she was admitted with HIV inaugural infection (CDC stage C3) and cerebral toxoplasmosis. As the first line of treatment, the patient was started on sulfadiazine and pyrimethamine^1,2,3^. After one week, she developed non-oliguric acute kidney injury. Urinary sediment analysis revealed sulfonamide crystals with the morphologic appearance of shocks of wheat (Figures 1 and 2), confirmed by infrared spectroscopy^4^. Sulfadiazine was replaced with clindamycin, and a notable enhancement was observed after to the implementation of vigorous fluid hydration using an alkaline solution (sodium bicarbonate).
