# Supplementary material for: Sulfadiazine-induced crystalluria and non-oliguric renal failure in HIV-1 inaugural infection with presumed cerebral toxoplasmosis
Source: J Bras Nefrol. 2024 May 10;46(4):e20230151. doi: 10.1590/2175-8239-JBN-2023-0151en (PMC11287978; doi:10.1590/2175-8239-JBN-2023-0151en)
Supplement: Translation

# Translation: Cristalúria induzida por sulfadiazina e insuficiência renal não oligúrica em infecção inaugural por HIV-1 com toxoplasmose cerebral presumida

**DOI:** 10.1590/2175-8239-JBN-2023-0151pt

**Authors:** Vasco Gaspar, Nuno Moreira Fonseca, Sara Lino

**Affiliations:** 1 Centro Hospital Universitário Lisboa Central, Departamento de Medicina Interna, Lisboa, Portugal.; 2 Universidade NOVA de Lisboa, Faculdade de Medicina NOVA, Lisboa, Portugal.; 3 Centro Hospitalar Universitário Lisboa Central, Hospital Curry Cabral, Departamento de Nefrologia, Lisboa, Portugal.; 4 Centro Hospitalar Universitário Lisboa Central, Hospital Curry Cabral, Departamento de Infecciologia, Lisboa, Portugal.

Uma mulher de 30 anos, sem histórico médico prévio, foi trazida ao departamento de emergência por convulsão e agressividade. Após extenso estudo analítico e imagiológico, ela foi internada por infecção inaugural por HIV (estágio C3 do CDC) e toxoplasmose cerebral. Como primeira linha de tratamento, foi iniciado sulfadiazina e pirimetamina^1,2,3^. Após uma semana, desenvolveu lesão renal aguda não oligúrica. A análise do sedimento urinário revelou cristais de sulfonamida com aspecto morfológico de feixes de trigo (Figuras 1 e 2), confirmados por espectroscopia de infravermelho^4^. A sulfadiazina foi substituída por clindamicina, e observou-se uma melhora significativa após a implementação de hidratação vigorosa com solução alcalina (bicarbonato de sódio).

**Figura 1:** Os cristais de sulfadiazina apresentam uma coloração âmbar e estrias radiais (microscopia de contraste de fase, ampliação de 400×). Resultados da análise urinária – densidade: 1,008; pH: 5; proteínas: 15 mg/dL; hemoglobina: 0,75 mg/dL; nitritos/glicose/cetonas/bilirrubina/urobilinogênio: negativo.

**Figura 2:** Os cristais de sulfadiazina são fortemente birrefringentes sob luz polarizada (luz polarizada, ampliação de 400×).
